# miR‐34a in extracellular vesicles from bone marrow mesenchymal stem cells reduces rheumatoid arthritis inflammation via the cyclin I/ATM/ATR/p53 axis

**DOI:** 10.1111/jcmm.15857

**Published:** 2021-01-19

**Authors:** Huaiguo Wu, Xike Zhou, Xuedong Wang, Wei Cheng, Xinjia Hu, Yueping Wang, Bing Luo, Wenjun Huang, Juan Gu

**Affiliations:** ^1^ Center for Precision Medicine Anhui No. 2 Provincial People’s Hospital Hefei China; ^2^ Department of Medical Laboratory Science The Fifth People’s Hospital of Wuxi, Nanjing Medical University Wuxi China; ^3^ Department of Pathology The Fifth People’s Hospital of Wuxi, The Medical School of Jiangnan University Wuxi China; ^4^ Department of Osteoarthropathy Shenzhen People’s Hospital, The Second Clinical Medical College of Jinan University and the First Affiliated Hospital of Southern University of Science and Technology Shenzhen China; ^5^ Department of Biology College of Arts & Science Massachusetts University Boston MA USA

**Keywords:** ATM/ATR/p53 signalling pathway, Cyclin I, extracellular vesicles, inflammation, MicroRNA‐34a, rheumatoid arthritis

## Abstract

Extracellular vesicles (Evs) participate in the development of rheumatoid arthritis (RA), but the mechanisms remain unclear. This study aimed to determine the mechanism by which microRNA‐34a (miR‐34a) contained in bone marrow mesenchymal stem cell (BM‐MSC)‐derived Evs functions in RA fibroblast‐like synoviocytes (RA‐FLSs). BM‐MSC‐derived Evs and an Evs inhibitor were extracted. A rat model of RA was established. miR‐34a gain‐ and loss‐of‐function experiments were performed, and the inflammation in rat synovial fluid and tissues was detected. The role of miR‐34a in RA‐FLSs was also measured in vitro. The target gene of miR‐34a was predicted using the online software TargetScan and identified using a dual‐luciferase reporter gene assay, and the activation of the ATM/ATR/p53 signalling pathway was assessed. BM‐MSC‐derived Evs mainly elevated miR‐34a expression, which reduced RA inflammation in vivo and inhibited RA‐FLS proliferation and resistance to apoptosis in vitro, while inhibited miR‐34a expression enhanced RA development. In addition, miR‐34a could target cyclin I to activate the ATM/ATR/p53 signalling pathway, thus inhibiting abnormal RA‐FLS growth and RA inflammation. Our study showed that miR‐34a contained in BM‐MSC‐derived Evs could reduce RA inflammation by inhibiting the cyclin I/ATM/ATR/p53 signalling pathway.

## 1 INTRODUCTION

Rheumatoid arthritis (RA) is one of the most commonly diagnosed systemic autoimmune diseases worldwide, and it can affect several organ systems.^1^, ^2^ Persistent inflammation often contributes to erosive joint injury and dysfunction in most RA patients.^3^ In addition to the inconvenience caused by the disease itself, RA patients also have an increased risk of comorbidities, including cardiovascular diseases, osteoporosis, myocardial infarction and malignancies; thus, RA patients have decreased quality of life and shortened life expectancy.^4^ Approximately half of the risk of the occurrence of RA is caused by genetic factors,^5^ and female gender and smoking are also risk factors for RA.^2^ Currently, disease‐modifying anti‐rheumatic drugs (DMARDs), such as methotrexate and sulfasalazine that can decrease joint inflammation and improve overall function, are the primary treatments for RA.^6^ In addition, the infiltration of T or B cells and macrophages and the secretion of inflammatory cytokines, including tumour necrosis factor‐α (TNF‐α) and interleukins (ILs), are observed in the synovial tissues of RA patients.^7^ Biologic treatments that target these cytokines have enhanced the outcomes of RA patients who show little response to conventional DMARDs.^8^ However, approximately 50% of RA patients do not respond to anti‐TNF‐α treatments, and patients whose inflammation is controlled cannot have their bone erosions repaired.^9^ Thus, other novel effective therapies are urgently needed.

Recently, human multipotent mesenchymal stem cells (MSCs) have attracted wide‐ranging interest as a novel therapy for cartilage defects, and their therapeutic potential has been shown to involve the secretion of extracellular vesicles (Evs).^10^ Evs are heterogeneous, small vesicles that include three common types: exosomes, microparticles and apoptotic bodies.^11^ Evs are known for their function in intercellular communication and their ability to transport their contents to cells and cause multiple changes in cell proliferation and cell transcription.^12^ Evs can modulate many types of pathological and physiological processes, including inflammation and immune responses, tumour growth and infection.^13^ Ev‐based therapies possess promising potential for inflammatory and autoimmune diseases and tumours.^14^, ^15^ Evs are now known to transfer microRNAs (miRs) between cells, transmitting their miRs to target cells through endocytosis or membrane fusion.^16^ It has been demonstrated that miRs in Evs can be transferred and function in immune cells, cancer cells and endothelial cells.^17^ miRs exhibit tissue‐specific expression patterns and are linked to various biological events, including cell growth, differentiation, apoptosis, cancer progression and autoimmune arthritis.^18^ Deregulation of miR expression has been extensively observed in human diseases, including autoinflammatory and autoimmune diseases.^19^ Jian et al demonstrated that elevation of miR‐34a can alleviate microglial inflammation and inhibit neuronal apoptosis, thus improving nerve recovery and motor function.^20^ In the current study, we examined the mechanism of bone marrow MSC (BM‐MSC)‐derived Evs in the development of RA and the involvement of miR‐34a and its downstream pathway.

## 2 MATERIALS AND METHODS

### 2.1 BM‐MSC culture

BM‐MSCs were purchased from the Cell Center of the Chinese Academy of Medical Sciences (Beijing, China) and incubated in Dulbecco's modified Eagle's medium (DMEM) containing 10% foetal bovine serum (FBS). After labelling with mouse anti‐human CD90‐fluorescein isothiocyanate (FITC), CD44‐FITC, CD73‐FITC, CD105‐FITC, CD34‐PE, CD14‐PE, CD45‐PC7 and HLA‐DR (all purchased from Abcam Inc, Cambridge, MA, USA), the BM‐MSCs were analysed by flow cytometry. Then, the BM‐MSCs were identified using OriCellTM assay kits (Cyageb Biosciences, Guangzhou, Guangdong, China) by Alizarin Red staining and Oil Red O staining to determine their abilities to differentiate into osteoblasts and adipocytes.

### 2.2 BM‐MSC‐derived Evs extraction

Well‐established BM‐MSC cultures of passage 3 were washed 3 times with sterile phosphate‐buffered saline (PBS), and then, the remaining medium was discarded and serum‐free medium was added. After incubation at 37°C for 24 hours, the supernatant was collected and considered the conditioned medium. Next, the medium was successively centrifuged at 300 g for 10 minutes, 2000 g for 30 minutes and 10 000 g for 1 hour, and then, the Evs were extracted after the cell debris and large granules were excluded via a 0.22‐µm filter. The conditioned medium from cells supplemented with GW4869, an EV inhibitor, was used as a control. Then, the protein concentration of the Evs was evaluated using a bicinchoninic acid (BCA) assay kit, and then, the Evs were frozen at −80°C.^21^ The extracted Evs were labelled using PKH26 (MIDI26‐1KT; Sigma‐Aldrich Chemical Company, St Louis, MO, USA). In addition, well‐established BM‐MSC cultures were transfected with the miR‐negative control (NC) and miR‐34a inhibitor (miR‐IN) (all purchased from GenePharma, Shanghai, China) and named the NC group and IN group, respectively. Forty‐eight hours later, the Evs in the BM‐MSCs were extracted and named the Evs‐NC and Evs‐IN, respectively.

Next, the Evs were resuspended and dropped onto a sealing film and observed under an 80‐kV transmission electron microscope (TEM). The levels of the Evs marker proteins CD63, CD81 and calnexin were detected using Western blot analysis, and the particle sizes of the Evs were measured using nanoparticle tracking analysis.

### 2.3 Establishment of the rat model of RA

A total of 30 male Wistar rats (150 ± 20 g in weight; 6 weeks old) were purchased from the Laboratory Animal Center of Chinese Academy of Sciences and were separately housed in class II clean rooms with free access to standard feed and water. A week later, 20 rats were randomly selected, and each of the rats was intradermally injected with Freund's complete adjuvant F5881 (Sigma‐Aldrich Chemical Company) through the left hindlimb toes at a dose of 0.1 mL/100 g; these rats were then assigned to the model group. The remaining 10 rats were injected with equal amounts of normal saline and assigned to the normal group.

After establishing the model, 5 rats in the model group were injected with 75 μg/mL Evs (Evs group), 5 rats in the NC group were injected with GW4869 as a negative control via the tail vein, 5 rats in the model group were injected with 75 μg/mL Evs‐NC (as Evs‐NC group), and 5 rats in the Evs‐IN group were injected with Evs‐IN. Then, the rat tissues were isolated, and the location of the Evs in the tissues was observed under a fluorescence microscope.

### 2.4 Evaluation of rat foot swelling

The volumes of the rat right hindlimb toes (non‐injection side) were measured one day before injection and at weeks 1, 2, 3 and 4 after injection. The degree of foot swelling was measured as follows: degree of swelling = average volume after injection ‐ average volume before injection.

### 2.5 Scoring of the polyarthritis index

The arthropathy of the rats was observed and recorded at weeks 1, 2, 3 and 4 after injection, and the polyarthritis index was evaluated with the following scores: 0 = no swelling; 1 = swelling of toes; 3 = swelling of foot claws below the ankle; 4 = swelling of all foot claws including the ankle. The total scores of both limbs were calculated as the polyarthritis index, and the maximum value was 12.^21^

### 2.6 Evaluation of the spleen and thymus indexes

Four weeks after establishing the model, the rats were killed, and their spleen and thymus were extracted and weighed. The index of the spleen (or thymus) of the rats was calculated as follows: spleen (or thymus) index = spleen weight (or thymus weight)/body weight. Then, the synovial tissues and synovial fluid of the rats were extracted. The synovial fluid was used for enzyme‐linked immunosorbent assay (ELISA), and the synovial tissues of the right hindlimb were used for section generation and staining, such as haematoxylin and eosin (HE) staining, immunohistochemistry and immunofluorescence staining, whereas the synovial tissues of the left hindlimb were used for quantitative real‐time polymerase chain reaction (qRT‐PCR), Western blot analysis and RA fibroblast‐like synoviocyte (RA‐FLS) separation.

### 2.7 HE staining

The extracted synovial tissues were fixed with 10% formalin for 24 hours, decalcified with 10% ethylenediaminetetraacetic acid (EDTA) for 30 minutes, dehydrated, embedded in paraffin and subjected to HE staining. Histological changes were observed under an optical microscope.

### 2.8 Immunohistochemistry

The tissue sections were fixed with 4% paraformaldehyde at 4°C for 30 minutes and then at room temperature for 10 minutes. Then, the sections were treated with 3% H_2_O_2_ for 15 minutes to inactivate the endogenous peroxidase, washed with 10% goat serum 3 times and sealed for 20 minutes at room temperature. Next, the sections were incubated with a caspase‐3 antibody (1:50, Abcam, Cambridge, MA, USA) at 4°C overnight. The second day, the sections were successively incubated with biotin‐labelled goat anti‐rabbit and horseradish peroxidase (HRP)‐labelled avidin at 37°C for 30 minutes. Next, the sections were treated with 2,4‐diaminobutyric acid (DAB, ZSGB‐BIO, Beijing, China), counterstained with haematoxylin, dehydrated with xylene, treated with alcohols and finally sealed with neutral balsam.

### 2.9 ELISA

The expression of IL‐6, IL‐1β and TNF‐α in the rat synovial fluid was detected using ELISA kits (Shanghai Bogoo Biological Technology Co., Ltd., Shanghai, China). The sandwich approach was applied during the assay as per the instructions of the kits.

### 2.10 qRT‐PCR

Total RNA was extracted from the synovial tissues using RNAiso Plus (Takara Holdings Inc, Kyoto, Japan) and TRIzol LS Reagent (Takara). Formaldehyde denaturing gel electrophoresis was used to determine the RNA quality for the subsequent experiments. Next, RNA reverse transcription PCR (RT‐PCR) was conducted strictly according to the instructions of the Prime Script^TM^ RT assay kit (Takara). The quantification of the mRNA and miR levels was conducted using SYBR Premix Ex Taq (Takara)‐based qRT‐PCR with glyceraldehyde‐3‐phosphate dehydrogenase (GAPDH) as the internal mRNA reference and U6 as the internal miR reference. The amplification curve and dissolution curve were confirmed after the reaction. The relative expression of the genes was calculated by the 2^−ΔΔCt^ method. The primer sequences are shown in Table 1.

**TABLE 1:** Primer sequences for qRT‐PCR

Gene	Sequence
miR‐34a	F: 5'‐TGGCAGTGTCTTAGCTGGTTGT‐3'
	R: 5'‐CTCAACTGGTGTCGTGGAGT‐3'
miR‐1180	F: 5'‐AACTACCTGGACCGCTTCCT‐3'
	R: 5'‐CCACTTGAGCTTGTTCACCA‐3'
miR‐123b	F: 5'‐GAAGGGGACCAACAGCTGGTTGA‐3'
	R: 5'‐AGCTGTTGGTCCCCTTCAACCAGC‐3'
TNF‐α	F: 5'‐AGCCCATGTTGTAGCAAACCCTC‐3'
	R: 5'‐TGGTTATCTCTCAGCTCCACGCCA‐3'
IL‐6	F: 5'‐TGCATCCCAGAACTAGACGTGC‐3'
	R: 5'‐TGCATCCCAGAACTAGACGTGC‐3'
IL‐8	F: 5'‐GAGAGTGATTGAGAGTGGACCAC‐3'
	R: 5'‐CACAACCCTCTGCACCCAGTTT‐3'
cyclin I	F: 5'‐CGCTCGAGCCACCATGAAGTTT‐3'
	R: 5'‐TCGAGCTACATGACAGAAAC‐3'
ATM	F: 5'‐TTGCCACACTCTTTCCATGT‐3'
	R: 5'‐CCCACTGCATATTCCTCCAT‐3'
ATR	F: 5'‐GATGTTCCTCTCGTTGTGGAG‐3'
	R: 5'‐CAGTGCAGTTAAGAGCCTTCC‐3'
p53	F: 5'‐ACCTGCACTTACTCCCCG‐3'
	R: 5'‐TCTTATAGACGGCCACGGCG‐3'
U6	F: 5'‐GCGCGTCGTGAAGCGTTC‐3'
	R: 5'‐GTGCAGGGTCCGAGGT‐3'
GAPDH	F: 5'‐CGGACCAATACGACCAA‐3'
	R: 5'‐AGCCACATCGCTCAGACACC‐3'

Abbreviations: ATM, ataxia‐telangiectasia mutated; ATR, ATM‐Rad3‐related; F, forward; GAPDH, glyceraldehyde‐3‐phosphate dehydrogenase; IL, interleukin; miR, microRNA; qRT‐PCR, quantitative real‐time polymerase chain reaction; R, reverse; TNF‐α, tumour necrosis factor‐α.

### 2.11 Western blot analysis

Total proteins were extracted using radioimmunoprecipitation assay (RIPA) buffer containing phenylmethylsulfonyl fluoride (PMSF, Beyotime Biotechnology Co., Ltd, Shanghai, China), and the protein level in the supernatant was confirmed using a BCA assay. Next, equal volumes of proteins (50 mg) were separated by 10% sodium dodecyl sulphate‐polyacrylamide gel electrophoresis and transferred to polyvinylidene difluoride (PVDF) membranes (Millipore Corp, Billerica, MA, USA). Then, the membranes were successively incubated with tris‐buffered saline tween (TBST, Boster Biological Technology Co., Ltd, Wuhan, Hubei, China) containing 5% skim milk to block non‐specific binding and then incubated with the indicated primary antibodies (all purchased from Abcam) (Table 2) at 4°C overnight. After the membranes were incubated with rat anti‐mouse antibodies at room temperature for 1 hour, the bands were visualized using enhanced chemiluminescence and imaged using a BioSpectrum system (Bio‐Rad, Inc, Hercules, CA, USA).

**TABLE 2:** Antibodies used in Western blot analysis

Antibodies	Cat. No.	Dilution ratio
β‐actin	ab179467	1:5000
CD81	ab79559	1:500
CD63	ab59479	1:1000
Tsg101	ab125011	1:1000
Calnexin	ab29559	1:100
PUMA	ab9643	1:50
Bax	ab32503	1:5000
Cleaved PARP	ab32064	1:5000
Cyclin I	ab192239	1:5000
ATM	ab81292,	1:50 000
ATR	ab2905	1:1000
P53	ab32389	1:1000

Abbreviations: ATM, ataxia‐telangiectasia mutated; ATR, ATM‐Rad3‐related; PARP, poly (ADP‐ribose) polymerases.

### 2.12 Rat synoviocyte separation and culture

Rat synoviocytes were extracted from the tissues using trypsinization. When the synoviocytes covered more than 80% of the bottom of the culture flask, they were passaged; the cells were passaged a total of three times. The cells harvested from the normal rats were assigned to the FLS group, whereas those from the model rats were assigned to the RA‐FLS group. FLSs and RA‐FLSs in the logarithmic growth phase were suspended using DMEM containing 10% FBS, seeded into 24‐well plates at a density of 1 × 10^5^ cells per well and cultivated in a 37℃ incubator with 5% CO_2_ and 95% humidity for 48 hours.

Well‐established RA‐FLS cultures were treated with 75 μg/mL BM‐MSC‐derived Evs, Evs‐NC or Evs‐IN and assigned to the Ev group, Evs‐NC group and Evs‐IN group, respectively. RA‐FLSs treated with BM‐MSCs supplemented with GW4869 were used as the NC group. After 36 hours of incubation, the RA‐FLSs were collected for the following experiments.

The cells in the Evs‐IN group were treated with the p53‐specific activator Tenovin‐6 (0.5 μg/mL) or an equal volume of PBS for 48 hours and then collected for further use.

### 2.13 RNA sequencing

The miRNAs of the cells in the RA‐FLS group and RA‐FLS‐Ev group were extracted using a miRcute miRNA isolation assay kit (DP150, Beijing TianGen Biotech Co., Ltd. Beijing, China). The sequences of three samples from each group were determined by Shanghai Sangon Biotech Co., Ltd. (Shanghai, China). The differentially expressed miRNAs in the RA‐FLS cells before and after EV treatment were screened, and a fold change >2 and *P* < 0.05 served as the screening criteria.

### 2.14 Immunofluorescence assay

Slides of cells in each group were fixed with 4% paraformaldehyde at 4°C for 15 minutes, treated with 0.5% Triton X‐100 for 20 minutes and then incubated with primary antibodies (all purchased from Abcam) (Table 3) at 4°C overnight. The next day, the cell slides were washed with PBS, incubated with goat anti‐rabbit at 37°C for 1 hour and then observed under a fluorescence microscope (Leica DM 3000, Solms, Germany).

**TABLE 3:** Antibodies used in immunofluorescence assay

Antibodies	Cat. No.	Dilution ratio
Vimentin	ab193555	1:1000
PCNA	ab92552	1:100
CD90	ab225	1:100
Cytochrome C	ab133504	1:000

Abbreviation: PCNA, proliferation cell nuclear antigen.

### 2.15 3‐(4,5‐Dimethylthiazol‐2‐yl)‐2,5‐diphenyltetrazolium bromide (MTT) assay

The viability of the cells in each group was detected strictly according to the instructions of the MTT assay kit (C0009, Beyotime Biotechnology Co., Ltd, Shanghai, China), and the optical density (OD) value was measured at 490 nm.

### 2.16 Flow cytometry analysis of Annexin V‐FITC/propidium iodide (PI) staining

Cell proliferation: After washing with PBS and detachment with 0.025% trypsin, the cells in each group were cultured in DMEM containing 2 mmoL carboxyfluorescein diacetate succinimidyl ester (CFSE) (Invitrogen Inc, Carlsbad, CA, USA) for 15‐30 minutes. Then, the cells were further washed 3 times with PBS, centrifuged, resuspended in DMEM and analysed by flow cytometry.

Cell cycle: The cell suspension was treated with −20°C 70% methanol (v/v) for 24 hours and then treated with 50 μg/mL PI containing 10 μg/mL RNase for 20 minutes. Then, the cell cycle distribution was measured.

Cell apoptosis: The cell suspension was stained with Annexin V‐FITC (5 μL) and PI (5 μL) in the dark for 10 minutes. Then, cell apoptosis was detected.

### 2.17 Dual‐luciferase reporter gene assay

A computer‐based miR target detection program was used to predict the binding sites of miR‐34a and cyclin I (http://www.targetscan.org/vert_72/). Then, pMIR‐REPORT^TM^‐based luciferase reporter plasmids (Thermo Fisher Scientific, CA, USA) containing wild‐type (WT) cyclin I or cyclin I that was mutated (MT) at the putative miR‐34a binding sites were designed by Shanghai Sangon Biotech Co., Ltd. (Shanghai, China). Well‐designed plasmids along with miR‐34a mimics and miR‐NC were cotransfected into 293T cells using Lipofectamine^TM^ 2000 (Invitrogen Inc, Carlsbad, CA, USA). The relative luciferase activity was determined using the dual‐luciferase reporter assay system according to the kit's instructions (Promega Corporation, WI, USA).

### 2.18 Statistical analysis

The Statistical Package for the Social Sciences (SPSS) 21.0 (IBM Corp. Armonk, NY, USA) was used for data analysis. The normality test was conducted using the Kolmogorov‐Smirnov method. The measurement data were normally distributed and expressed as the mean ± standard deviation. Differences between pairs of groups were evaluated using the *t* test, whereas differences among multiple groups were compared using one‐way analysis of variance (ANOVA). Tukey's multiple comparisons test was used for pairwise comparisons after ANOVA. The *P* value was calculated using a two‐sided test, and *P* < 0.05 was considered to show a statistically significant difference.

## 3 RESULTS

### 3.1 Identification of BM‐MSCs and BM‐MSC‐derived Evs

**FIGURE 1:**
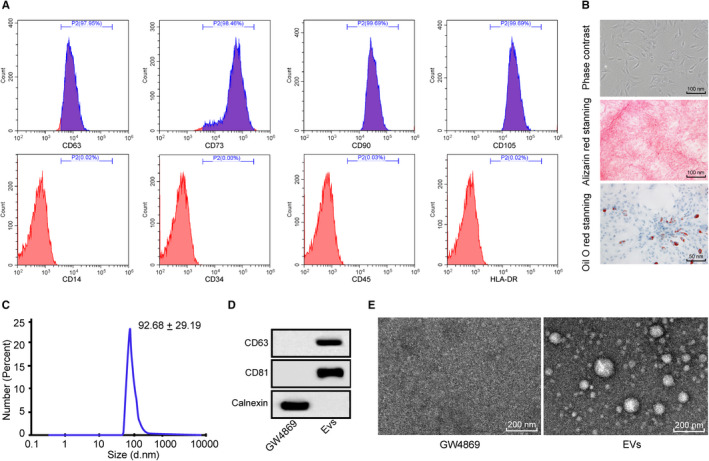
Identification of BM‐MSCs and BM‐MSC‐derived Evs. A, Biomarkers of BM‐MSCs detected by flow cytometry; B, morphological observation of BM‐MSCs, and differentiation ability of BM‐MSCs measured with Alizarin Red staining and Oil Red O staining; C, sizes of Evs measured using nanoparticle tracking analysis; D, Evs‐related proteins evaluated using Western blot analysis, with the cells grown in conditioned medium supplemented with GW4869, an EV inhibitor, as a control; E, Evs morphology and size observed by TEM, with the cells grown in conditioned medium supplemented with GW4869, an EV inhibitor, as a control. BM‐MSCs: bone marrow mesenchymal stem cells; Evs: extracellular vesicles; TEM: transmission electron microscope

To identify the quality of BM‐MSCs, we detected biomarkers of BM‐MSCs and found that the cells were positive for CD44, CD73, CD90 and CD105, whereas the cells were negative for CD14, CD34, CD45 and HLA‐DR (Figure 1A). The Alizarin Red staining and Oil Red O staining results suggested that the BM‐MSCs were able to differentiate into osteoblasts and adipocytes (Figure 1B). Thus, the BM‐MSCs qualified for the following experiments.

The size of the Evs was identified by a nanoparticle tracker, and the results showed that the diameter of the Evs was approximately 93 nm (Figure 1C). The protein levels of the Ev markers CD63, CD81 and calnexin were measured by Western blot analysis. The results suggested that the Evs were positive for CD63 and CD81, whereas the Evs were negative for calnexin (Figure 1D). According to the TEM observations, the extracted Evs were uniformly distributed with different sizes (Figure 1E).

### 3.2 BM‐MSC‐derived Evs reduce inflammation in rats with RA

**FIGURE 2:**
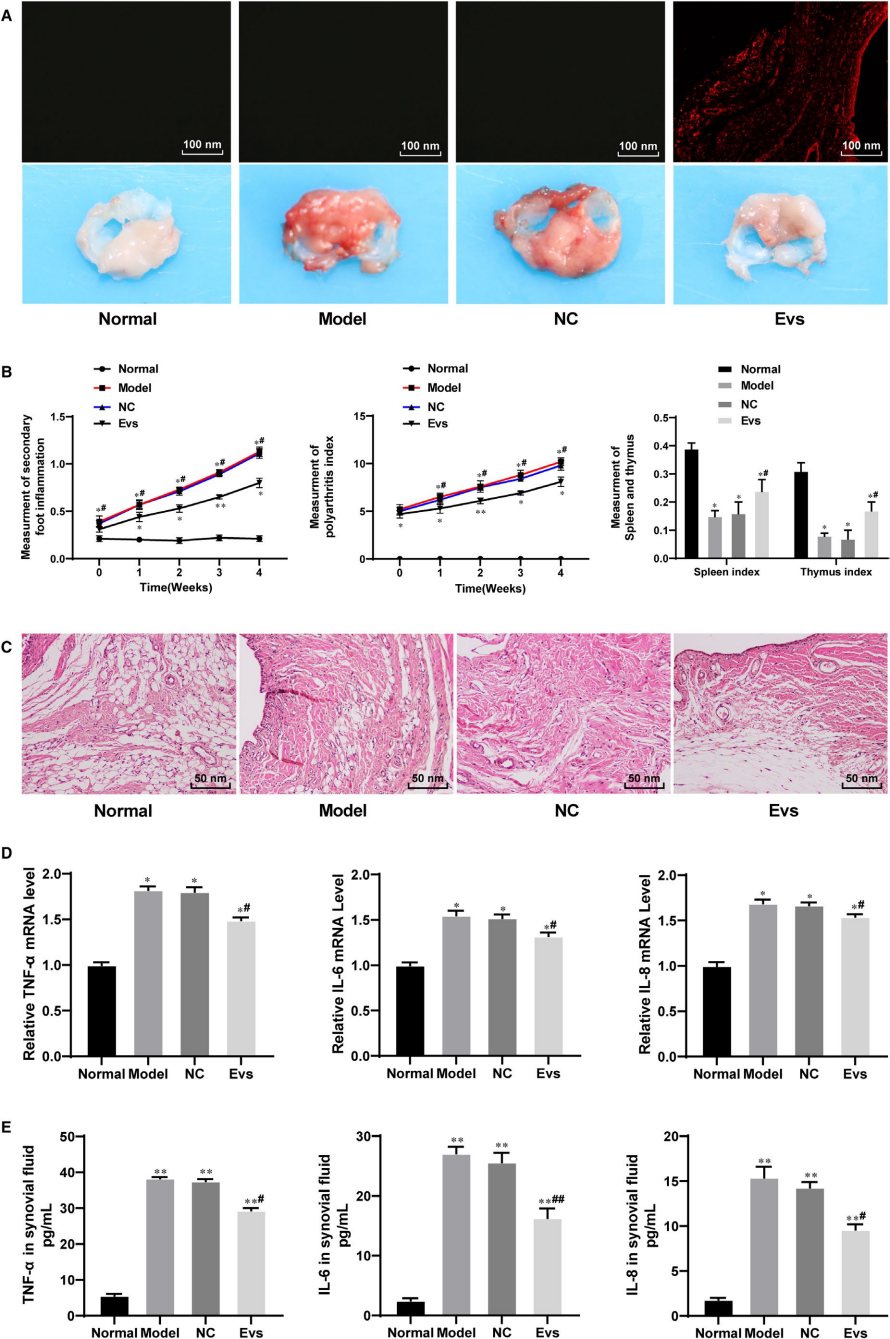
BM‐MSC‐derived Evs reduce inflammation in RA rats. A, Immunofluorescence of PKH26 for the absorption of PKH26‐labelled EVs by synovium; B, evaluation of RA inflammation in rats; C, the morphology of synovium observed using HE staining; D&E, expression of TNF‐α, IL‐6 and IL‐8 in rat synovial tissues detected using qRT‐PCR (D) and in synovial fluid detected using ELISA (E); n = 3. Compared with the normal group; *P < 0.05, **P < 0.01; compared with the model group; ^#^ *P* < 0.05, ^##^ *P* < 0.01. The normal group represented the rats injected with saline; the model group represented the rats with RA; the NC group represented the rats injected with BM‐MSC medium containing GW4869; and the EV group represented the RA rats injected with EVs. RA: rheumatoid arthritis

To explore the role of Evs derived from BM‐MSCs in RA, we injected 75 μg/mL Evs (Ev group) into the tail vein of model rats and injected GW4869 as a control (NC group). The Evs extracted from BM‐MSCs were labelled with PKH26, and based on the immunofluorescence of PKH26, we found that the Evs were transferred into the synovial tissues (Figure 2A). The degree of swelling in the feet of the normal rats and RA rats was obviously enhanced, whereas the degree of swelling in the feet of the RA rats injected with Evs was reduced (Figure 2B). HE staining showed obvious synovial hyperplasia and inflammatory infiltration in the model and normal rats, whereas synovial hyperplasia and inflammatory infiltration in the rats treated with Evs were significantly reduced compared with those in the model rats (Figure 2C). Moreover, the levels of TNF‐α, IL‐6 and IL‐8 were notably higher in the synovial tissues and synovial fluid from the RA rats than in those from the normal rats, while these factors were significantly decreased in the RA rats after Ev injection (Figure 2D‐E).

### 3.3 BM‐MSC‐derived Evs inhibit RA‐FLS proliferation

**FIGURE 3:**
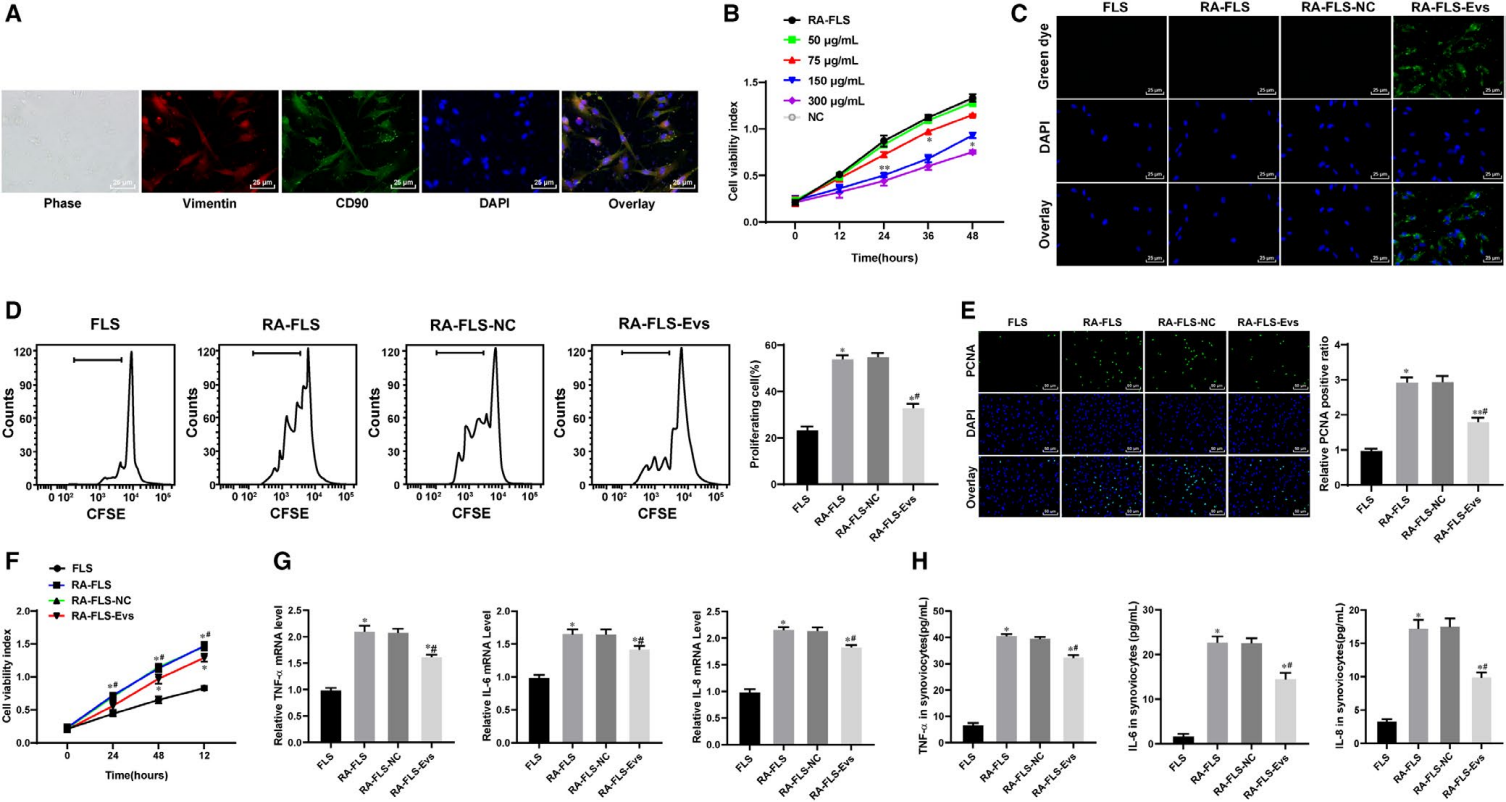
BM‐MSC‐derived Evs inhibit RA‐FLS proliferation and inflammation. A, Immunofluorescence of CD90 and Vimentin; B, dose and time determination of Ev treatment measured by MTT assay; C, immunofluorescence of PKH26 for the absorption of PKH26‐labelled EVs by FLSs; D, cell proliferation detected using flow cytometry; E, cell viability detected using MTT assay; F, immunofluorescence of PCNA; G&H, expression of TNF‐α, IL‐6 and IL‐8 in cells detected using qRT‐PCR (G) and ELISA (H); n = 3; compared with the normal group; *P < 0.05, **P < 0.01; compared with the RA‐FLS group; ^#^ *P* < 0.05, ^##^ *P* < 0.01. FLSs were cultured from the synovial tissues of rats in the normal group, whereas RA‐FLSs were cultured from the synovial tissues of rats in the model group. RA‐FLS‐NC are RA‐FLS cells grown in BM‐MSC medium supplemented with GW4869, whereas RA‐FLS‐Evs were RA‐FLS cells to which Evs were added. FLS: fibroblast‐like synoviocytes

To verify the experimental results in vivo, we named the cells from the synovium of the normal rats FLSs and the cells cultured from the synovium of the model rats RA‐FLSs. The immunofluorescence of CD90 and Vimentin showed that the extracted cells were FLSs (Figure 3A). The best dose and time of treatment of FLSs with Evs were 75 μg/mL and 36 hours, as indicated by the MTT assay (Figure 3B). Moreover, the PKH‐26‐labelled Evs acted on RA‐FLSs, and the immunofluorescence suggested that the Evs were absorbed by the RA‐FLSs (Figure 3C). According to the results of flow cytometry, MTT and immunofluorescence assays, the cell proliferation ability, cell viability and proliferation cell nuclear antigen (PCNA) positive rate were significantly enhanced in RA‐FLSs compared with FLSs, while treatment with Evs reversed these changes (Figure 3D‐F). Then, the levels of TNF‐α, IL‐6 and IL‐8 were detected by RT‐qPCR and ELISA. The results showed that the mRNA and protein levels of TNF‐α, IL‐6 and IL‐8 in the RA‐FLS group were significantly higher than those in the FLS group, and the expression of inflammatory factors was inhibited by Ev treatment (Figure 3G‐H).

### 3.4 BM‐MSC‐derived Evs mainly increase miR‐34a expression

**FIGURE 4:**
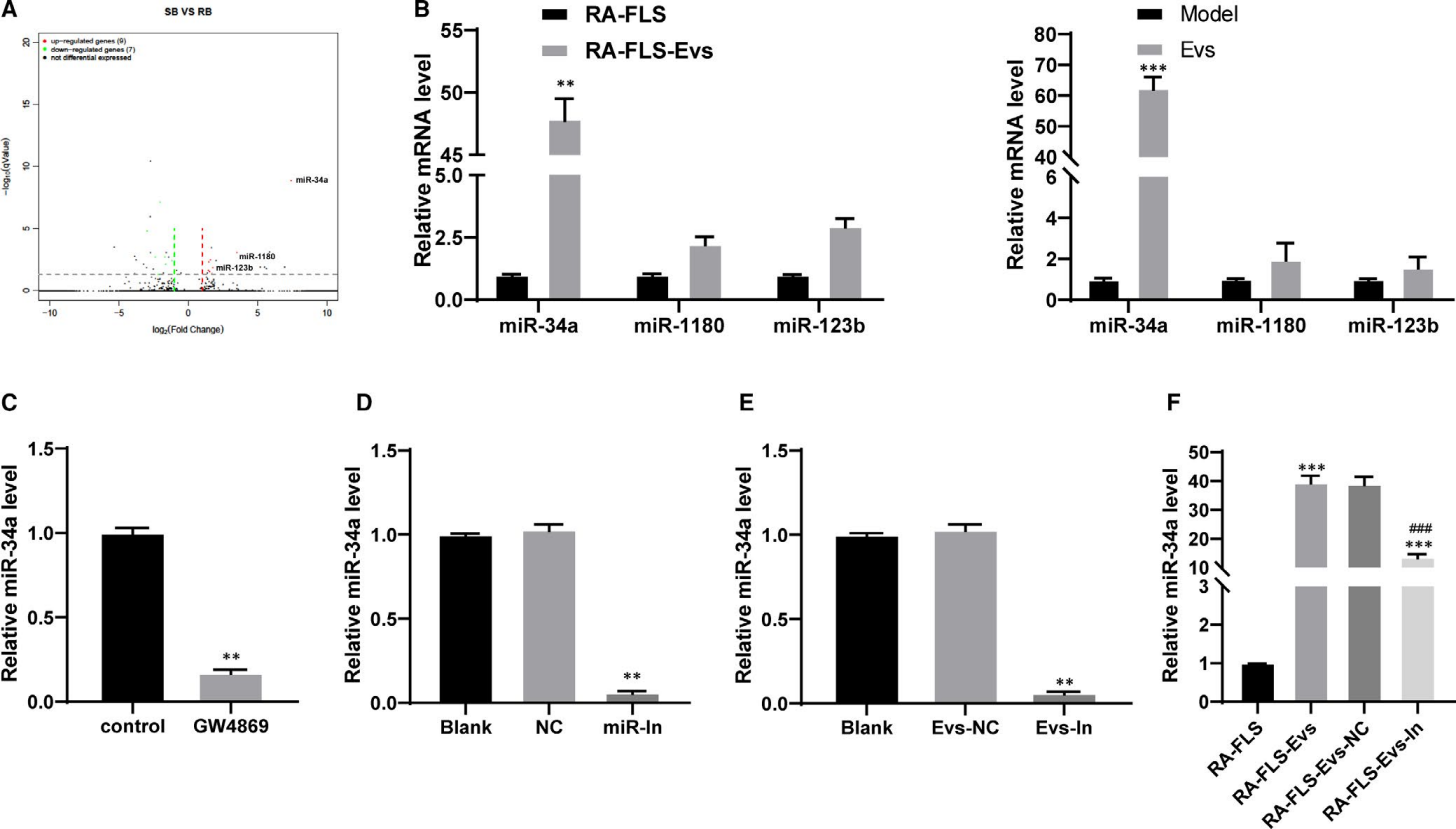
BM‐MSC‐derived Evs mainly elevate miR‐34a expression. A, volcano plots of miRNAs whose expression changed after Evs treatment; B, miR‐34a, miR‐1180 and miR123b expression in cells after Evs treatment detected using qRT‐PCR, * compared with the RA‐FLS or model group; C, miR‐34a expression in BM‐MSCs after sphingomyelinase inhibitor (GW4869) treatment, * compared with the control group; D, miR‐34a expression in BM‐MSCs detected using qRT‐PCR, * compared with the NC group; E, miR‐34a expression in Evs measured using qRT‐PCR, * compared with the Evs‐NC group; F, miR‐34a expression in RA‐FLS after different Evs treatment measured using qRT‐PCR, *compared to RA‐FLSs, #compared with RA‐FLS‐Evs; n = 3; *P < 0.05, **P < 0.01, *** or ^###^ *P* < 0.001. miR‐In: miR‐34a inhibitor; Evs‐In: Evs transfected with miR‐34a inhibitor

EVs play a vital role in intercellular communication and regulate recipient cells by transferring their contents, including proteins and nucleic acids such as miRs.^22^ Based on the results described above that BM‐MSC‐derived Evs induce RA inflammation, the focus shifted to determining the potential molecular mechanisms involved in these effects. RNA sequencing was used to evaluate the differentially expressed miRNAs between the RA‐FLSs before and after Ev treatment and the untreated RA‐FLSs. A total of 16 miRNAs showed differential expression, with 9 miRNAs up‐regulated and 7 miRNAs down‐regulated (Figure 4A). Among the 9 miRNAs enhanced in the FLS‐Evs, the most differentially expressed miRNA was miR‐34a, the log_2_ fold change of which was 7.69 (Table 4). In addition, the expression of miR‐34a, miR‐1180 and miR‐123b in the RA‐FLSs, RA‐FLS‐Evs and tissues of the model group and Ev group was detected using qRT‐PCR, and the results suggested that the expression of miR‐34a changed the most, and the expression of miR‐34a exhibited the same trend as the RNA sequencing results (Figure 4B).

**TABLE 4:** microRNAs whose expression elevated in Evs

Gene name	log_2_FoldChange	*P* value
miR‐34a	7.69	4.39E−06
miR‐1180	4.42	7.50E−15
miR‐123b	3.51	3.39E−08
miR‐138‐5P	1.77	1.29E−06
miR‐146a	1.70	4.97E−06
miR‐214	1.61	1.82E−07
miR‐199b‐3P	1.54	2.73E−06
miR‐335a	1.48	3.22E−07
miR‐449a	1.44	2.40E−06

Abbreviations: Evs, extracellular vesicles; miR, microRNA.

To further identify the correlation between Evs and miR‐34a expression, we treated BM‐MSCs with a sphingomyelinase inhibitor (GW4869) to suppress Ev production and then detected miR‐34a expression in the cells. The results showed that miR‐34a mainly existed in Evs, and inhibition of Evs led to inhibition of miR‐34a expression (Figure 4C).

BM‐MSCs were transfected with an miR‐34a inhibitor or miR‐NC using a Lipofectamine^TM^ 2000 kit. Next, qRT‐PCR confirmed the transfection (Figure 4D). After the extraction of Evs, the expression of miR‐34a in the Evs was detected. After inhibiting the expression of miR‐34a, the expression of miR‐34a in the Evs significantly decreased (Figure 4E). The concentration of Evs was adjusted to 75 μg/ mL, and the EVs were added to the RA‐FLSs. miR‐34a expression in the cells treated with RA‐FLS‐Evs‐IN was significantly lower than that in the cells treated with RA‐FLS‐Evs (Figure 4F), indicating that miR‐34a was carried into cells by Evs.

### 3.5 Reduction of miR‐34a in BM‐MSC‐derived Evs weakens the protective role of BM‐MSC‐derived Evs in rats with RA

**FIGURE 5:**
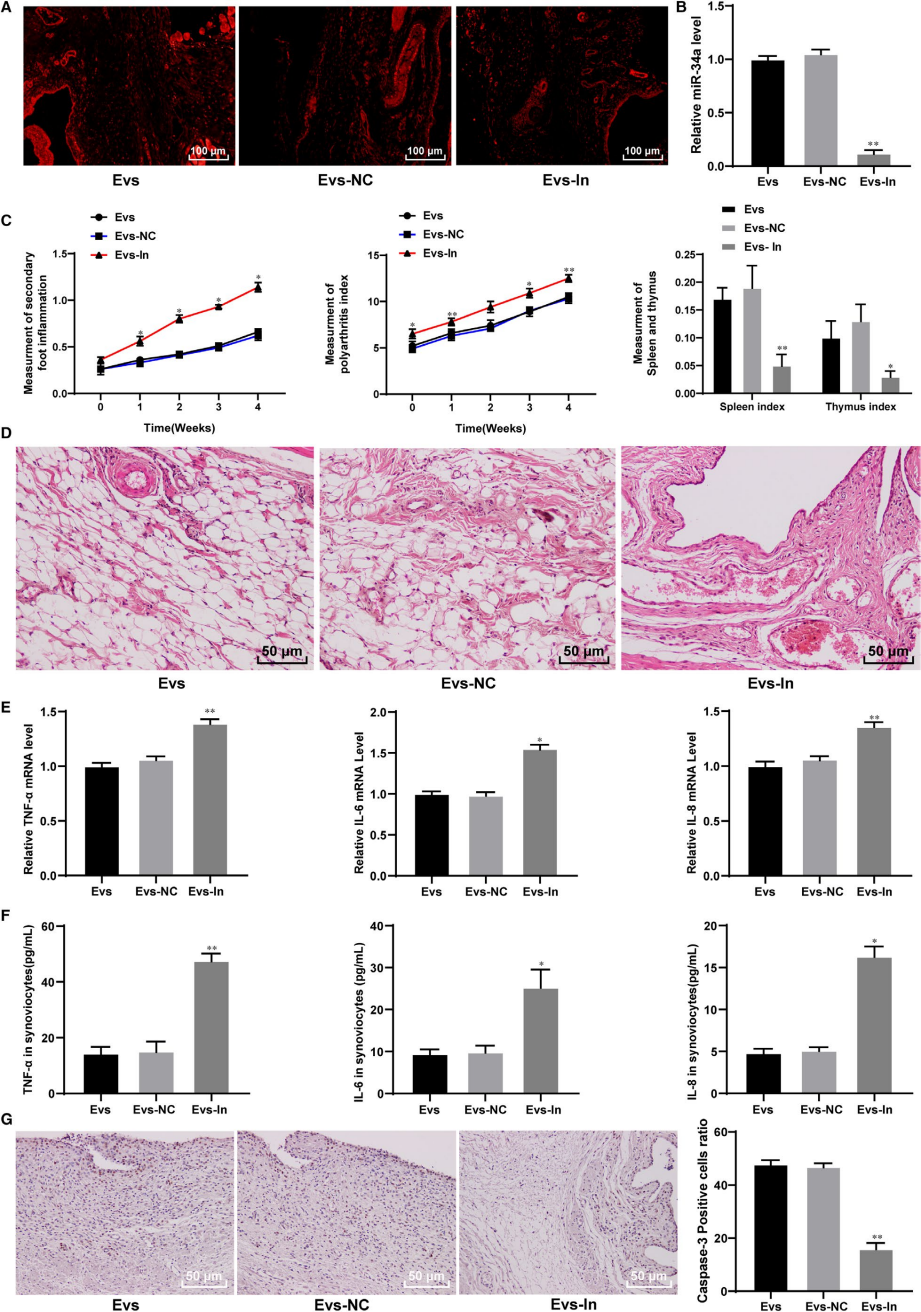
miR‐34a suppresses inflammation in RA rats. A, immunofluorescence of PKH26 in synovial tissues; B, miR‐34a expression in synovial tissues detected using qRT‐PCR; C, evaluation of RA inflammation in rats; D, inflammation detected using HE staining; E&F, expression of TNF‐α, IL‐6 and IL‐8 in rat synovial tissues detected using qRT‐PCR (E) and synovial fluid detected using ELISA (F); G, caspase‐3 expression measured using immunohistochemistry; n = 3; *compared with the NC group; *P < 0.05, **P < 0.01

Rats with RA were treated with Evs, Evs‐NC or Evs‐IN, and the immunofluorescence of PKH‐26 suggested that the Evs were absorbed by the synovial tissues (Figure 5A). Next, qRT‐PCR confirmed the transfection (Figure 5B). The level of miR‐34a in the EVs‐IN group was significantly lower than that in the Evs‐NC group. Compared with that in the Evs‐NC group, the degree of swelling of the lateral feet in the Evs‐IN group was significantly increased (Figure 5C). The HE staining results showed that compared with that in the Evs‐NC group, the level of inflammation of the synovial tissue in the EV‐treated group significantly increased (Figure 5D). In addition, the mRNA (Figure 5E) and protein levels (Figure 5F) of TNF‐α, IL‐6 and IL‐8 in the tissues showed the same trend. The results of caspase‐3 immunohistochemistry showed that after inhibiting miR‐34a expression, positive caspase‐3 expression was decreased (Figure 5G).

### 3.6 miR‐34a contained in BM‐MSC‐derived Evs inhibits RA‐FLS proliferation and induces RA‐FLS apoptosis

**FIGURE 6:**
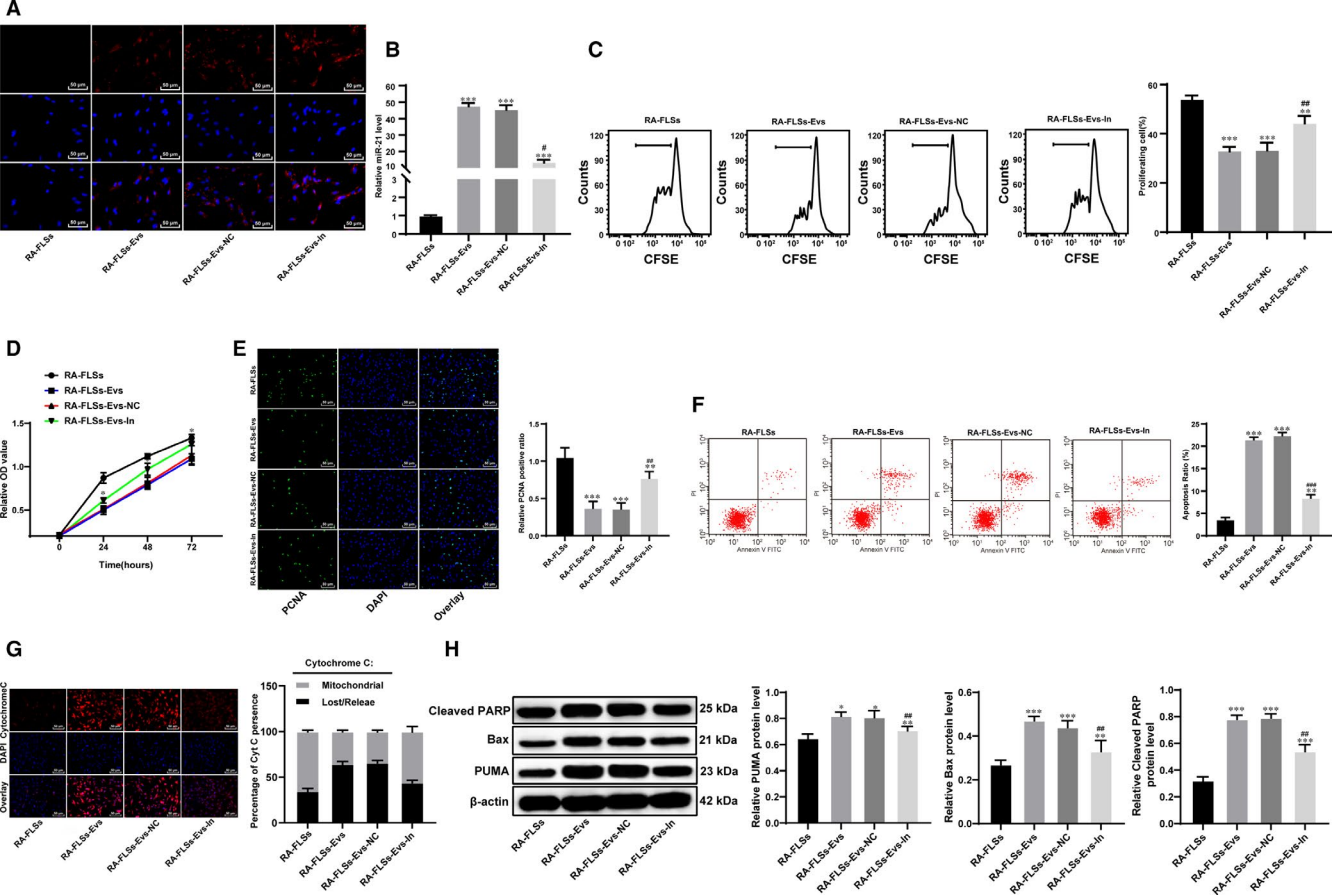
miR‐34a inhibits RA‐FLS proliferation and promotes RA‐FLS apoptosis. A, Immunohistochemistry of PKH26 in RA‐FLSs; B, miR‐34a expression in RA‐FLSs detected using qRT‐PCR; C, cell proliferation detected using flow cytometry; D, cell viability detected using MTT assay; E, immunofluorescence of PCNA; F, flow cytometry used to detect apoptosis; G, immunofluorescence of cytochrome C; H, expression of Bax, PUMA and cleaved PARP detected using Western blot analysis, *compared with the blank group; n = 3; *P < 0.05, **P < 0.01; compared with the Evs group, #*P* < 0.05, ## *P* < 0.01, ###*P* < 0.001

Immunofluorescence of PKH26, a specific marker of EVs, showed that Evs in the Ev group were absorbed by RA‐FLSs (Figure 6A). The qRT‐PCR results suggested that miR‐34a expression was significantly reduced in RA‐FLSs transfected with Evs‐IN (Figure 6B). The low expression of miR‐34a in BM‐MSC‐derived Evs enhanced the proliferation, viability and positive PCNA rate of the RA‐FLSs (Figure 6C‐E).

The flow cytometry, cytochrome C immunohistochemistry and Western blot analysis results suggested that inhibiting miR‐34a expression in BM‐MSC‐derived Evs reduced the cell apoptosis rate, prevented cytochrome 3 from transferring to the cytoplasm and reduced the expression of apoptosis‐related proteins, including Bax, PUMA and cleaved poly (ADP‐ribose) polymerase (PARP) (Figure 6F‐I).

### 3.7 miR‐34a targets cyclin I and activates the ATM/ATR/p53 signalling pathway

**FIGURE 7:**
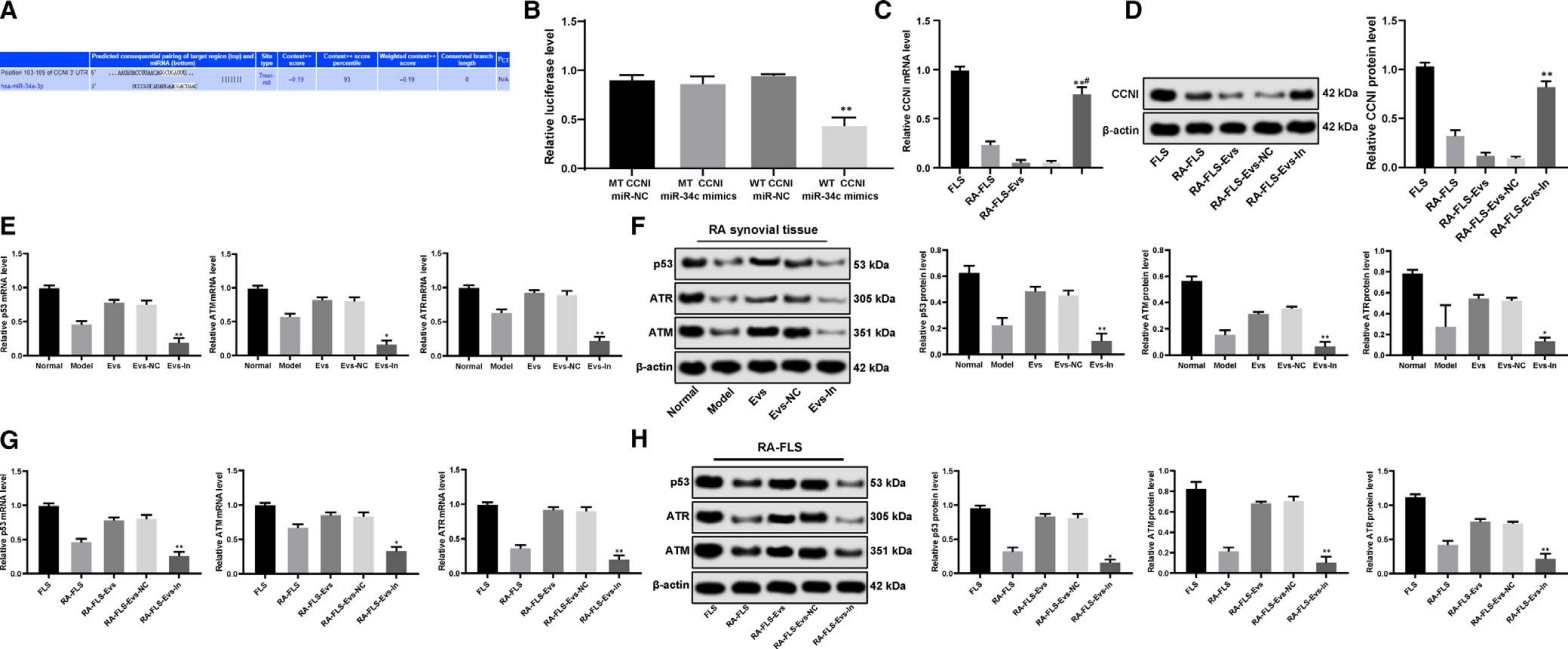
miR‐34a targets cyclin I and activates the ATM/ATR/p53 signalling pathway. A, Prediction of the target gene of miR‐34a by TargetScan; B, identification of miR‐34a binding cyclin I using a dual‐luciferase reporter gene assay; C&D, mRNA and protein expression of cyclin I in cells detected using qRT‐PCR (C) and Western blot analysis (D); E&F, ATM, ATR and p53 expression in the synovial tissues of the RA rats; G&H, ATM, ATR and p53 expression in FLSs; *compared with the NC group, *P < 0.05, **P < 0.01; n = 3. ATM: ataxia‐telangiectasia mutated; ATR: ataxia‐telangiectasia and Rad3‐related

The computer‐based miR target detection program (http://www.targetscan.org) predicted that miR‐34a could directly bind to the 3' UTR of cyclin I (Figure 7A). The binding relationship was also identified using a dual‐luciferase reporter gene assay. It was shown that the relative luciferase activity showed little difference between the MT + miR mimics group and the MT + miR‐NC group, but the luciferase activity was notably reduced in the cells transfected with WT + miR mimics (*P* < 0.01) (Figure 7B).

Moreover, the qRT‐PCR and Western blot analysis results revealed that the mRNA and protein expression of cyclin I in the cells treated with Evs‐IN was significantly enhanced compared to that in the cells treated with Evs (*P* < 0.05) (Figure 7C‐D).

In addition, the association of miR‐34a and the ATM/ATR/p53 signalling pathway was determined. qRT‐PCR and Western blot analysis were used to detect the expression of p53, ATM and ATR in RA‐FLSs, and the results demonstrated that silencing miR‐34a in BM‐MSC‐derived Evs led to reduced p53, ATM and ATR expression both in rat synovial tissues and RA‐FLSs (Figure 7E‐H).

Hence, these results showed that miR‐34a could negatively target cyclin I and then activate the ATM/ATR/p53 signalling pathway, thus inhibiting cell proliferation and cell apoptosis resistance.

### 3.8 Cyclin I inactivates the ATM/ATR/p53 signalling pathway

**FIGURE 8:**
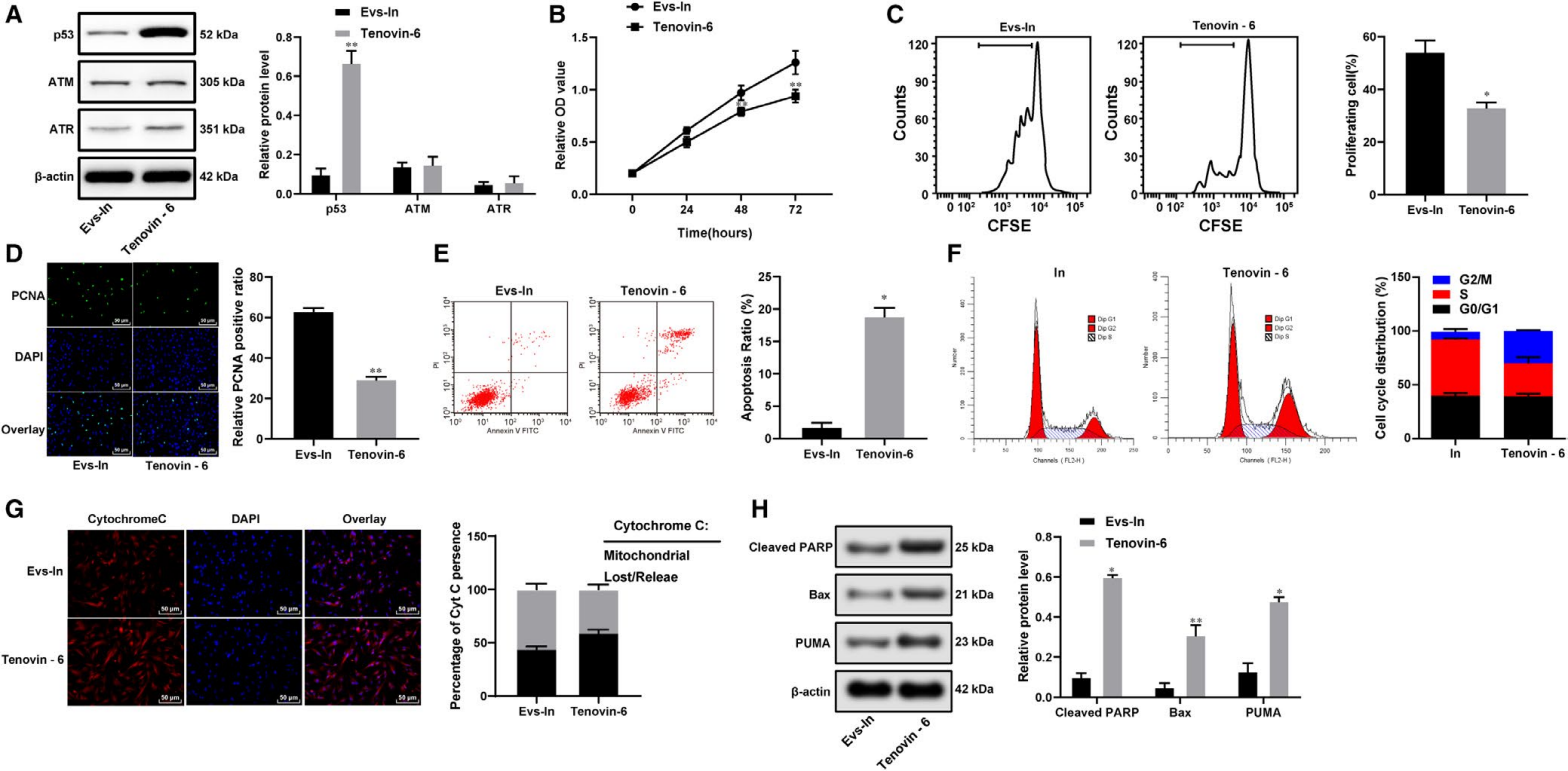
Cyclin I inactivates the ATM/ATR/p53 signalling pathway. A, Expression of ATM, ATR and p53 in RA‐FLSs after Tenovin‐6 treatment; B, RA‐FLS viability after Tenovin‐6 treatment detected with MTT assay; C, RA‐FLS proliferation detected using flow cytometry; D, immunofluorescence of PCNA; E, FLS apoptosis detected using flow cytometry; F, cell cycle distribution evaluated using flow cytometry; G, immunofluorescence of cytochrome C; H, apoptosis‐related protein detected using Western blot analysis; n = 3; *compared with the Evs‐IN group; *P < 0.05, **P < 0.01

To further determine the role of cyclin I in the development of RA, the RA‐FLSs in the Evs‐IN group were treated with the p53‐specific activator Tenovin‐6 for rescue experiments. The results revealed that after Tenovin‐6 treatment, p53 was activated **(**Figure 8A), and the cell proliferation, viability and resistance to apoptosis and cell cycle arrest of the RA‐FLSs were significantly reduced compared to those of the Evs‐IN‐treated RA‐FLSs (Figure 8B‐H). All the results described above showed that miR‐34a contained in BM‐MSC‐derived Evs could inhibit cyclin I expression to activate the ATM/ATR/p53 signalling pathway, thus regulating the function of synovial fibroblasts.

## 4 DISCUSSION

RA is a common chronic autoimmune disease that leads to articular destruction and related vascular, metabolic and bone comorbidities.^23^ Evs have been recognized as key mediators of intercellular communication, and EVs are capable of transmitting biological signals between cells and then regulating diverse biological processes.^15^ Evs can carry a variety of miRNAs.^24^ In this study, we established a rat model of RA to examine the biological mechanisms by which BM‐MSC‐extracted Evs participate in the inflammation of RA and found that miR‐34a from BM‐MSC‐derived Evs could regulate the function of synovial fibroblasts in RA by inhibiting cyclin I and activating the ATM/ATR/p53 signalling pathway.

Initially, the RA model rats showed obviously enhanced limb swelling, increased FLS numbers and promoted inflammation in synovial tissues, and TNF‐α, IL‐6 and IL‐8 expression was notably enhanced both in vivo and in vitro; however, BM‐MSC‐derived Ev transfection reversed these symptoms in the RA model rats. Evs are implicated in the progression of RA, playing critical roles in antigen release, angiogenesis, inflammation and intercellular signal communication.^9^ MSC‐derived Evs could reduce inflammation in RA due to their immunosuppressive, antifibrotic and anti‐inflammatory functions.^11^ Importantly, our study found that BM‐MSC‐derived Evs inhibited RA‐FLS proliferation and viability and decreased PCNA expression. PCNA provides a molecular platform that facilitates various processes in the maintenance and duplication of the genome.^25^ FLSs are well‐known resident mesenchymal cells in synovial joints whose activation leads to the production of a large amount of soluble mediators that retain and activate resident joint cells and immune system cells, thus promoting inflammation and tissue destruction.^26^ FLSs develop specific immune properties during RA development and show an activated, harmful phenotype in the synovium.^27^ TNF‐α is an inflammatory promoter involved in the pathogenesis of multiple human diseases.^28^ IL‐6 and IL‐8 are two common inflammatory mediators that are implicated in RA patients, and these mediators play key roles in the regulation of the acute phase response and contribute to neutrophil infiltration into the synovial fluid in RA patients.^29^ The activation of FLSs and TNF‐α activates proinflammatory cytokines to promote inflammation and resistance to apoptosis in T cells.^12^

The anti‐inflammatory role of BM‐MSC‐derived Evs prompted us to further investigate the mechanisms involved in this event. Importantly, the in vitro study suggested that miR‐34a is the most abundant miR in BM‐MSC‐derived Evs. Inhibition of miR‐34a led to significantly enhanced inflammation but decreased caspase‐3 expression in RA rats, and it led to enhanced proliferation of RA‐FLSs and decreased expression of cytochrome C, Bax, PUMA and cleaved PARP in RA‐FLSs. miR‐34a is a well‐recognized potential tumour suppressor, and its anti‐inflammatory attributes have been shown.^30^ Moreover, miR‐34a has been suggested to enhance the expression of apoptosis‐related factors, including PUMA, thus leading to apoptosis.^31^ Caspase‐3, cytochrome C, Bax, PUMA and cleaved PARP are well‐recognized apoptotic‐related factors.^32^, ^33^ The secretion of cytochrome C further activates downstream caspases, such as caspase‐3 and cleaved PARP, to initiate cell apoptosis.^34^ Moreover, our study showed that miR‐34a could directly bind to cyclin I. It has been suggested that miR‐34a could target another member of the cyclin family, namely cyclin D1, in a previous study.^35^ The suppression of miR‐34a significantly elevated the expression of cyclin I and reduced the expression of p53, ATM and ATR in synovial tissues and RA‐FLSs. miR‐34a knockdown aggravated inflammation (elevated levels of TNF‐α and IL‐6), inhibited cell proliferation and enhanced apoptosis in an in vitro model of spinal cord injury.^36^ Cyclin I is an atypical cyclin known for its prosurvival and anti‐apoptosis functions by mediating the activation of cyclin‐dependent kinases (CDKs),^37^ while ATM/ATR is a cell cycle checkpoint signalling pathway whose activation could lead to cell cycle arrest and cell apoptosis by inactivating CDKs.^38^, ^39^ Furthermore, a previous study showed that the induction of cyclin D1 could inhibit the phosphorylation of ATM/ATR targets, thus inactivating the ATM/ATR signalling pathway.^40^ Thus, we concluded that miR‐34a could negatively bind to cyclin I to activate the ATM/ATR/p53 signalling pathway, thus inhibiting the resistance of cell cycle arrest and reducing the inflammation of RA.

In summary, the current study provided evidence that miR‐34a contained in BM‐MSC‐derived Evs could bind to the 3' UTR of cyclin I and further enhance the activation of the ATM/ATR/p53 signalling pathway, thus promoting RA‐FLS apoptosis and reducing RA inflammation. These results provide novel insights into the development of new therapies for RA prevention. However, the present study is limited by a lack of experiments on the mechanism of miR‐34a in Evs. Additionally, we will further study the effect of human BM‐MSCs in a rat model of RA in the future. Hopefully, more studies will be carried out in this field to reveal more details on the role of BM‐MSC‐derived Evs in RA and to develop more strategies for RA prevention and treatment.

## CONFLICT OF INTEREST

The authors declare that they have no competing interests.

## AUTHOR CONTRIBUTIONS

**Xinjia Hu:** Conceptualization (lead); Project administration (equal); Writing‐original draft (lead); Writing‐review & editing (equal). **Xike Zhou:** Data curation (equal); Formal analysis (equal); Project administration (equal); Validation (equal). **Xuedong Wang:** Data curation (equal); Formal analysis (equal); Investigation (equal). **Wei Cheng:** Data curation (equal); Formal analysis (equal); Software (equal). **Huaiguo Wu:** Data curation (equal); Formal analysis (equal); Software (equal). **Yueping Wang:** Formal analysis (equal); Resources (equal); Software (equal). **Bing Luo:** Formal analysis (equal); Investigation (equal). **Wenjun Huang:** Writing‐original draft (equal); Writing‐review & editing (equal). **Juan Gu:** Writing‐original draft (equal); Writing‐review & editing (equal).

## ETHICAL STATEMENT

The current study was approved by the Clinical Ethical Committee of Shenzhen People's Hospital. All the experimental procedures were conducted in line with the ethical guidelines for studies involving experimental pain in conscious animals.

## Data Availability

All the data generated or analysed during this study are included in this published article.
